# Asymmetric effects of luminance and chrominance in the watercolor illusion

**DOI:** 10.3389/fnhum.2014.00723

**Published:** 2014-09-12

**Authors:** Andrew J. Coia, Michael A. Crognale

**Affiliations:** Department of Psychology, Cognitive and Brain Sciences, University of NevadaReno, NV, USA

**Keywords:** color vision, watercolor illusion, color spreading, contours, S cone pathways

## Abstract

When bounded by a line of sufficient contrast, the desaturated hue of a colored line will spread over an enclosed area, an effect known as the watercolor illusion. The contrast of the two lines can be in luminance, chromaticity, or a combination of both. The effect is most salient when the enclosing line has greater contrast with the background than the line that induces the spreading color. In most prior experiments with watercolor spreading, the luminance of both lines has been lower than the background. An achromatic version of the illusion exists where a dark line will spread while being bounded by either a darker or brighter line. In a previous study we measured the strength of the watercolor effect in which the colored inducing line was isoluminant to the background, and found an illusion for both brighter and darker achromatic outer contours. We also found the strength of spreading is stronger for bluish (+S cone input) colors compared to yellowish (−S cone input) ones, when bounded by a dark line. The current study set out to measure the hue dependence of the watercolor illusion when inducing colors are flanked with brighter (increment) as opposed to darker outer lines. The asymmetry in the watercolor effect with S cone input was enhanced when the inducing contrast was an increment rather than a decrement. Further experiments explored the relationship between the perceived contrast of these chromatic lines when paired with luminance increments and decrements and revealed that the perceived contrast of luminance increments and decrements is dependent on which isoluminant color they are paired with. In addition to known hue asymmetries in the watercolor illusion there are asymmetries between luminance increments and decrements that are also hue dependent. These latter asymmetries may be related to the perceived contrast of the hue/luminance parings.

## Introduction

The experience of color and brightness is not determined solely by the spectral content and number of photons and is instead influenced greatly by adaptive state and contextual features. Among the contextual effects are those that are produced by the presence of edges or contours. Several demonstrations have shown that when contour information is present, color spreading can be induced over a large spatial area. One example of color spreading is the watercolor illusion (e.g., Pinna, 1987; Pinna et al., 2001). The watercolor illusion consists of two adjacent contrasting lines forming a percept of “figure and ground” or enclosure. The figure and ground areas appear to be tinged with the coloration of the most proximal line. A governing principle of the watercolor effect is the asymmetric luminance contrast principle (Pinna, 2004). This states that for the watercolor illusion the color will spread more for a line that contrasts less with the background.

Most previous studies of the watercolor effect have used lines that contrast in both chromaticity and luminance. Devinck et al. quantified the magnitude of the watercolor effect by having observers match the perceived induction to a non inducing reference area (Devinck et al., 2005). They showed that similar results could be attained by applying an opposing hue over the induced area, cancelling out the illusion. With this method of hue cancellation they measured the illusion strength of different colors, bordered by their opponent counterparts as well as illusions with different luminance ratios of the contours. By dividing the amount of hue required to cancel the illusion by the amount of color in the inducing contour, they acquired a shift size which they used as an illusion magnitude. Their results indicated that most colors produced similar strength illusions, except yellow which was noticeably weaker. They also found that the strength of the effect increases as the luminance of the inner contour is increased towards the background. In a follow up study they showed that the illusion is also stronger the more chromatic contrast the two lines have (Devinck et al., 2006). Devinck et al. (2006) also looked at isoluminant chromatic watercolor illusions along different opponent axes and found L-M modulated illusions to be stronger than S modulated illusions.

An achromatic watercolor illusion can also be made by lines that contrast only in luminance (Cao et al., 2011). Cao et al. (2011) created a watercolor effect using a black inner line and achromatic outer lines spanning a wide range of luminances. They compared the induced darkness of the illusion to physically darkened control regions and found that for both brighter and darker outer lines the illusion strength was maximum at intermediate contrast levels. For their participants that perceived the illusion strongly, the effect was stronger for the opposite polarity condition, that is when the inner line was dark and the outer line was light. This observation for the achromatic watercolor effect is in agreement with the finding of Devinck et al. (2006) that increasing the chromatic contrast of the two contours increases the illusion strength. Furthermore, Cao et al.’s finding that intermediate luminance contrast levels produced the strongest illusions is consistent with Devinck et al.’s result that increasing colorimetric purity levels only increased the magnitude of the illusion to a certain extent.

Devinck et al. (2005) found that the illusion is weaker when the outer line is brighter than the inner line (on a brighter background) in agreement with the asymmetric contrast principle. However, Cao et al. (2011) showed that for achromatic spreading a brighter outer line induced equal if not stronger spreading for an inducing dark line on an intermediate background. Although one major difference between these studies is the induction being chromatic and achromatic, the difference in results likely arises from differences in the relative contrasts of the lines with the background. In Devinck et al. (2005) the lines where the inducing line was darker than the outer line were similar in luminance contrast to each other and both much darker than the background. In Cao et al. (2011), the background luminance was intermediate to those of the lines producing increments and decrements of contrast. It should be noted that despite these differences, both results are consistent with the asymmetric contrast principle.

It has been well established that when the background is the brightest part of the stimuli and the outer line is the darkest, increasing the luminance of the inner line and consequently reducing its contrast with the background will increase the magnitude of the illusion. Devinck and Knoblauch (2012) created a technique to measure the strength of the watercolor effect by showing a triad of illusions differing in inducing contour luminance and asking subjects which of the two bottom stimuli were more similar to the top reference. This avoids problems of methods such as having to distinguish between the coloration and figural effects which can be confounded by participants when faced with comparing the illusion to physical color.

The asymmetric luminance contrast principle along with results of these previous studies indicate that color spreading of the watercolor effect may be optimal when the inducing chromatic contour is isoluminant with the background, while the outer contour contrasts significantly in luminance/chrominance with the background and inducing line. Previously, Coia et al. (2014) examined the watercolor illusion with an inducing contour that was isoluminant with the background (and the outer contour differed from the background only in luminance). They showed that, for an orange inducing contour on an isoluminant background, a similar strength illusion occurred when the outer line was darker (decrement) or brighter (increment). They also measured the illusion strength of different hues in a cone opponent space with a dark outer line. In this study they equated the visibility of the different inducing contour hues using suprathreshold contrast matching (Switkes and Crognale, 1999; Switkes, 2008). This technique has been shown to produce accurate and reliable matches between luminance and chromatic patterns while obeying the principles of transitivity and homogeneity. Coia et al. (2014) found a larger effect for +S cone (violet) than for −S cone (yellowish) inducers and interpreted the finding as an asymmetry in illusion strength along the opponent S axis. These results are similar to those of Devinck et al. (2005), wherein most colors produced an approximately equal illusion, except for −S cone (lime/yellow), which was weaker than the others. In addition to the behavioral results Coia et al. also obtained supporting evidence for this asymmetry using the visual evoked potential (VEP).

**Figure 1:**
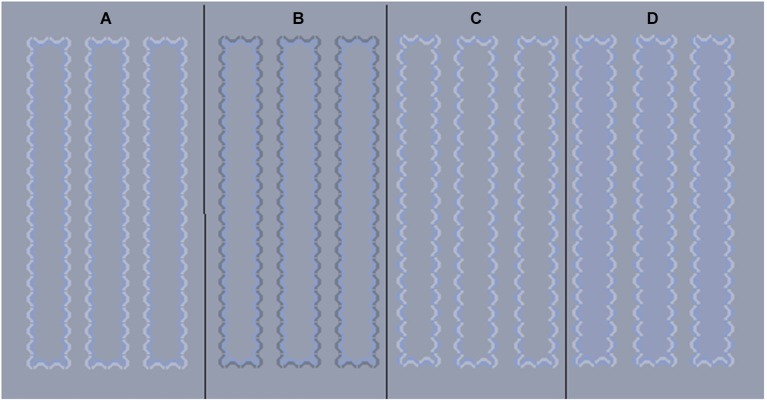
**Example stimuli. (A)** Illusion with blue inner line and white outer line, **(B)** Illusion with blue inner line and black outer line, **(C)** A blue/white control (no illusion) pattern that braids chromatic and achromatic contours, **(D)** same control pattern with physical color added to column interiors.

While much previous work has shown that the principle of asymmetric contrast applies under most conditions and that there is a hue dependence for the illusion strength, there is a question of whether or not luminance contrast increments and decrements produce similar hue-dependent effects (chromatic asymmetries). If the asymmetric contrast principle were the sole governing rule for strength of spreading, then luminance increments and decrements should produce equal magnitude effects as was shown previously for orange (Coia et al., 2014). It also follows that increments and decrements should produce similar hue-dependent asymmetries. We address this question by measuring the strength of the watercolor effect for different isoluminant hues in a cone opponent space using luminance increments (white line, Figure 1A) and decrements (Figure 1B). This revealed an enhancement to the chromatic asymmetry we had previously seen between +S cone (violet) and −S (lime) colors. In order to better understand these results, we conducted two additional contrast matching experiments wherein we examined the relationship between the apparent contrast of increments, decrements, and isoluminant hues. Experiment 2 tested the hypothesis that certain hue directions may require different amounts of contrast to match to luminance increments than decrements. Experiment 3 paired isoluminant contours with luminance increments and compared the resulting contrast to the same contours paired with luminance decrements.

## Experiment 1

We generated a watercolor illusion with an inner colored line that was isoluminant with the background and bordered by a lighter line (Figure 1A). Data using a darker outer line (Figure 1B) from Coia et al. (2014), were included for comparison. Eight different directions in MBDKL color space (MacLeod and Boynton, 1979; Derrington et al., 1984) were explored in this experiment, consisting of the four cardinal directions (+L−M (reddish), −L+M (greenish), +S (violet), −S (lime/yellow)) and four intermediate directions.

### Materials and methods

#### Participants

The experiments were conducted at the University of Nevada, Reno and were approved by the University’s Office of Human Research Protection. The majority of participants were undergraduate volunteers who gave informed consent to partake. All participants passed a color vision assessment consisting of the Ishihara test plates, Cambridge color test, and/or the Mollon-Reffin test. Before each experiment, a visual demonstration of the experiment was presented accompanied by verbal instructions.

Four students from the University of Nevada, Reno completed Experiment 1. All four students had also participated in a previous experiment with a dark outer line and had individually contrast matched the colors of the patterns.

#### Apparatus

All experiments were computer generated and displayed on a CRT monitor (Sony Multiscan 20 SE 2). The first experiment was conducted using a PC with a VSG2 graphics card (Cambridge) running Microsoft Visual Basic. The rest of the experiments were conducted using a PC running MATLAB and the Psychophysics Toolbox extensions (Brainard, 1997; Pelli, 1997). The monitor was calibrated using a PR650 spectrophotometer and an Optical photometer (Cambridge).

#### Stimuli

All experiments were done on a gray background (Commission Internationale de l’Eclairage (CIE) *x* = 0.310, *y* = 0.316). The stimuli (Figure 1) consisted of small arcs that were closely spaced forming column-like structures. The illusion is present when the inner arcs are all the same color and the outer arcs all a different color or luminance (Figures 1A,B). Controls in which the two colors are intertwined for inner and outer layers of pattern (Devinck and Knoblauch, 2012) were used in the contrast matching studies, minimum flicker experiments, and were used in a comparison to the illusion to estimate illusion strength (Figure 1C). At a viewing distance of 114 cm each arc subtended about 4.5 arcmin in width and the resulting columns were 0.66° s wide by 6.7° s long. Each group of illusion and control patterns comprised seven columns, spaced 0.37 degrees apart.

In order to get a measure of illusion strength, the illusion was compared to control columns that had 12 levels of saturation of a physical color inserted in the inner area (Figure 1D). This approach is similar to the one used by Cao et al. (2011) to study the achromatic affect. The chromaticities of these hues fell on a line in color space, between the white point and the inducing contour color. The background luminance was held at 18 cd/m^2^, as was the inner chromatic contour. The saturation of the inner contours and step sizes of inner control colors were the same as used previously to measure the illusion with a black outer line (Figure 1B) and are provided in the Table 1 (from Coia et al., 2014). The white line segments were held at a luminance of 27 cd/m^2^ (Weber contrast with background = 0.5) and the black line was 10 cd/m^2^ (Weber contrast with background = −0.44).

**Table 1:** **CIE chromaticity coordinates of the average endpoints of the axes used as inducing contours in experiment 1**.

**MBDKL angle**	**0 (+L−M)**	**27**	**90 (+S)**	**153**	**180 (+M−L)**	**207**	**270 (−S)**	**333**
CIE *x*	0.34	0.29	0.29	0.27	0.28	0.32	0.36	0.36
CIE *y*	0.36	0.24	0.26	0.27	0.33	0.38	0.42	0.38

*These values are an average of suprathreshold contrast matches across observers, and each observer used his/her custom matches for the endpoints along these axes*.

#### Procedure

Participants were seated in a dark room and completed a 2-alternative forced-choice (2AFC) paired comparison test comparing the illusory stimulus to a control pattern. The stimuli appeared on the screen for 250 ms and were followed by a blank screen. While fixating on a central fixation point, participants pressed a button on the keyboard corresponding to the side (left or right) that appeared to have more color on the inside of the columns. Each trial consisted of a presentation of the test pattern next to a control pattern filled with one of 12 randomly chosen saturation levels for each of the eight hues, counterbalancing position (left and right). This resulted in 192 trials for each run. Participants completed three runs in the experimental session.

### Results

**Figure 2:**
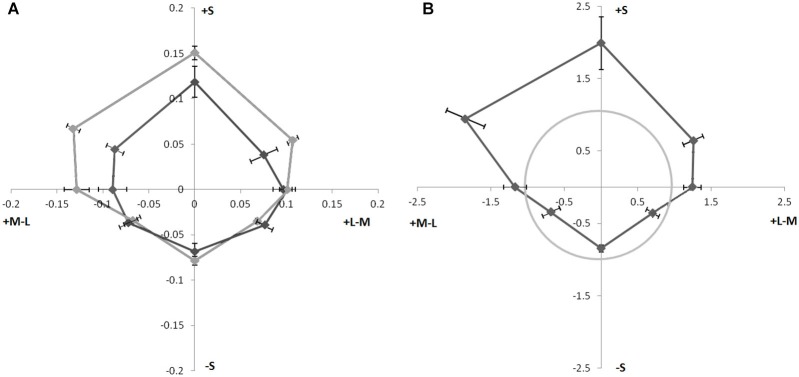
**(A)** Magnitude of watercolor illusion strength of colors with luminance increment (lighter line) and decrement (dark line, from Coia et al., 2014). Angle from origin represents color angle in MBDKL space. Distance from origin represents illusion magnitude as the fraction of the distance to the inducing contour. Error Bars ±1 SEM.** (B)** Ratio of increment/decrement illusion strength of different hues in isoluminant plane for four observers in the current study. Distance from the origin represents the ratio (increment/decrement) of the illusion magnitude, The gray circle indicates equal magnitude strength for luminance increments and decrements. Points outside of the circle had stronger illusion strength when bordered by a white line. Error Bars ±1 SEM.

The results were averaged and fit to psychometric functions (Weibull, 1951) with the saturation of the comparison hue that resulted in 50% choice of test vs. control taken as an estimate magnitude of the illusion. Values to express the illusion magnitudes were computed by dividing the distance between the background chromaticity and the chromaticity of the chosen saturation by the distance between the background chromaticity and the chromaticity of the inducing line. The illusion magnitude for increments and decrements (decrements from Coia et al., 2014) are shown in Figure 2A as polar plots in an opponent color space. Hue asymmetries can be seen under both conditions with +S stimuli producing the largest magnitude illusions. Another asymmetry can be seen between increments and decrements with increments producing larger illusion magnitudes than decrements for most hue directions, except the three −S cone directions in the lower quadrants. These hue directions seemed to not differ significantly when compared to the results from Coia et al. (2014; Figure 2A), and when compared to the decrement data for the four subjects alone appear to decrease slightly (Figure 2B).

The hue dependence of the illusion magnitude was analyzed using ANOVA on the increment and decrement data from Coia et al. (2014) separately since they were run in different experiments and had different numbers of subjects. Mauchly’s test of sphericity indicated that sphericity could not be assumed for either data set due to the unequal differences in variability between the different hues. Therefore we applied the conservative Greenhouse-Geisser correction that adjusts the degrees of freedom to both data sets (Greenhouse and Geisser, 1959). For the increment data there was a significant effect of hue direction; *F*_(1,2.18)_ = 11.80, *p* = 0.003. A *post hoc* *t*-test on the difference between the +Scone and −S cone hue directions revealed a significant difference between these magnitudes; *t*_(3)_ = 6; *p* = 0.009. The results from the decrement data from Coia et al. (2014) however, were marginally insignificant using the conservative Greenhouse-Geisser correction, due to large interobserver variability *F*_(1,3.718)_ = 2.537, *p* = 0.058. A similar *post hoc* *t*-test on the difference between the +S cone and −S cone magnitudes for these decrement data was significant, *t*_(11)_ = 2.73, *p* = 0.02.

The illusion strengths for increments and decrements were also directly compared by analyzing the data from the four subjects who had completed both increment and decrement tasks. The ratios of the magnitude of the illusions for increments vs. decrements was compared for each subject and hue direction. The ratios for each of the eight hues were then averaged across observers and plotted in the isoluminant plane of a cone-opponent color space (Figure 2B) as distance from the origin of the polar plot. If increments and decrements had produced equal strength illusions, then the data would plot at the value of 1 (gray circle). However, the illusion magnitude for +S color directions is enhanced when paired with a white outer line. A one-way repeated measure ANOVA was used to test for a main effect of hue in the ratios of illusion strengths for white/black outer lines. Because of violations of sphericity revealed by Mauchly’s test, we again adjusted our degrees of freedom according to the conservative Greenhouse-Geisser correction. There was a main effect in the ratio of lighter and darker illusion magnitudes for hue direction, *F*_(1,2.073)_ = 10.633, *p* = 0.01. A *post hoc* *t*-test comparing the results from the +S cone direction with those from the −S cone direction revealed a significant effect, *t*_(3)_ = 4,417, *p* = 0.022. These results suggest that the asymmetry along the S cone axis is enhanced when inducing hues are paired with luminance increments compared to when inducing hues are paired with luminance decrements.

## Experiment 2: Increment vs. decrement contrast matching

The luminance asymmetries revealed in the results of Experiment 1 raise a question about the source of this asymmetry. One possibility is that colors paired with increments spread more easily than colors paired with decrements even though the pairings have equal perceptual contrast. Another possibility is that the increase in illusion magnitude is actually driven by differences in perceived contrast of the pairings, with greater perceived contrasts resulting in greater induction of the color spreading. In addition, the perceived contrast may also show a hue/luminance interaction such that the increase in perceived contrast with increments is greater for some colors than it is for others. This possibility exists because the original contrast matching was done using inducing colors paired with black lines and it is possible that color matches pairing inducing colors with white lines would differ. If this is the case, then perhaps the illusion and the hue asymmetry was stronger with white lines only because the apparent contrast was greater, particularly along the +S cone axis. Experiment 2 tested the possibility that there may be a difference in contrast matches for achromatic contours of opposite luminance polarity. Note that this experiment measured the apparent contrast of the contours themselves and was not intended to produce a watercolor illusion.

### Methods

#### Participants

Eight students participated in Experiment 2.

#### Stimuli

In order to equate perceived contrast across different hues, participants matched the contrast of individual (unpaired) chromatic contours to that of individual (unpaired) standard achromatic contours. The contours were composed of arc segments and formed columns similar to those used in Experiment 1. The same eight directions in color space tested in Experiment 1 were used in Experiment 2. The background and the chromatic contour luminances were set to 10 cd/m^2^, while the luminances of the standard darker (decrement) and lighter (increment) achromatic contours were held at 9 and 11 cd/m^2^, respectively. The achromatic lines, although of low contrast with the background, were found in pilot studies to be appropriate for the task, allowing for suprathreshold contrast matches with the chromatic patterns. Each chromatic contour was compared to both incremental and decremental achromatic contours.

#### Procedure

Stimuli were presented in a 2AFC paradigm where chromatic columns were always compared to achromatic columns, using the method of constant stimuli (5 saturation levels). Participants were instructed to determine which set of columns was more visible. They were told to attend to the contours themselves, not the color of the inner area of the columns as in Experiment 1. There were eight colors, each having five levels of saturation that were compared with both black and white lines. Presentation side (left/right) was also counterbalanced for a total of 160 trials per run. They were initially presented with a sample stimulus and given a chance to perform a practice match.

### Results

**Figure 3:**
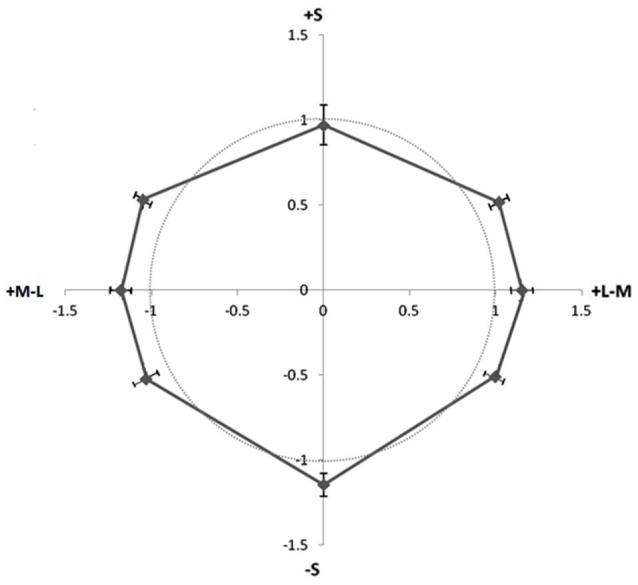
**Ratio of contrasts (increments/decrements) of unpaired luminance contours required to match unpaired isoluminant chromatic contours**. Different directions in color space represent different hues, and distance from origin represents the ratio of contrast matches (luminance increment match/luminance decrement match). Points outside circle required greater increment than decrement contrast to match the color contrast. Error Bars ±1 SEM.

All eight participants completed three runs. Their contrast matches were averaged and fit to psychometric functions. The 50% performance for picking color over luminance was taken as the matching contrast for each chromaticity. Each person’s matches to luminance increments were divided by their luminance decrements match for each of the eight colors. In Figure 3, the circle indicates a ratio of 1 where perceived contrast is equivalent for increments and decrements, and the points represent the ratio of chromatic contrast matches to luminance increments over matches to luminance decrements for each color direction. For the majority of colors (seven out of eight), more chromatic contrast was needed to match the white (luminance increment) line compared to black (points lying outside the circle in Figure 3), making their ratios greater than one. The +S direction on average has about an equal ratio, but also a higher variability across participants. An ANOVA revealed no significant differences in the increment/decrement ratios of colors, *F*_(1,7)_ = 1.046, *p* = 0.412. The results would predict that pairing a color with a lighter line might produce a stronger watercolor illusion than pairing the color with a darker line for all colors except +S cone. This is in contradiction to what was found in Experiment 1 wherein the advantage of increments over decrements was enhanced for +S cone hues. The results of Experiment 2 suggest that differences in the apparent contrast between the background and luminance increments and decrements by themselves cannot explain the difference in white/black illusion ratios found in Experiment 1 wherein colors with +S input had stronger illusions with increments than with decrements.

## Experiment 3: Increment vs. decrement contrast matching 2

The above results using unpaired contours suggest that luminance increments may be perceived as having a higher contrast with the background than luminance decrements. However, since there is no color dependence to this effect, the result cannot by itself explain an asymmetry as a function of color direction. To further address the hue asymmetry, we compared the apparent contrast of colored contours paired with lighter contours with the apparent contrast of those colors paired with darker contours. If the perceived color contrast using the white lines was greater than that produced using the black lines for colors with +S input, then this may explain the relative increase in magnitude of the illusion for these color directions.

### Methods

#### Participants

Seven students (different than those in Experiments 1 and 2) completed Experiment 3.

#### Stimuli

Stimuli consisted of a comparison of two patterns similar to the control patterns of Experiment 1, each having one half of their arcs chromatic and the other half achromatic. While the chromatic arcs were physically identical for both patterns, the achromatic arcs were various luminance increments or decrements. In one condition, the achromatic luminance increments were manipulated and the comparison decrements were held constant. In the second condition, the decrements were manipulated and compared with fixed increments. While the comparison luminance contours were held ±1 cd/m^2^ from the background, the test contours were presented at one of five levels ranging from 0.4–2 cd/m^2^ in the opposing luminance direction from the background (relative to the comparison pattern). The background for this experiment was 10 cd/m^2^. Chromatic contrasts were chosen using lab averages from contrast matching experiments. Individual isoluminance was determined using heterochromatic flicker photometry for each of the eight chromatic arcs. Colored arcs were then recombined with the achromatic arcs for the actual experimental trials.

#### Procedure

As in experiment 2, participants were shown a demonstration of the stimuli and instructed to choose which set of patterns (left or right) was more visible. We again used a 2AFC test with the method of constant stimuli. They were told to attend to the contours themselves, not the color of the inner area of the columns. Each run contained 160 trials: 8 colors × 5 luminance steps × 2 sides × 2 luminance comparisons.

### Results

**Figure 4:**
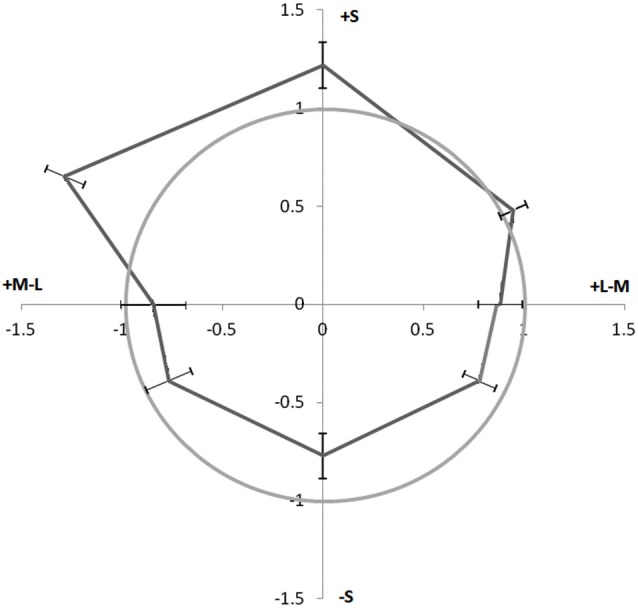
**Perceived contrast of chromatic contours paired with increments vs. decrements**. Perceived contrast is expressed as a ratio of increment over decrement sensitivity as a function of chromaticity. Distance from origin represents sensitivity ratio, and direction represents color. The gray circle represents an equal perceived contrast for pairings with decrements and increments. Points outside the gray circle have increased perceived contrast when paired with luminance increments. Error Bars ±1 SEM.

Participants completed three runs each. Data were sorted, averaged, and fit to psychometric (Weibull) functions. The 50% performance point was taken as the matching contrast for each chromaticity. The Weber contrast of these luminance matches (with respect to the background) was then divided by the Weber contrast of the fixed reference value in order to get a ratio of increment over decrement sensitivity. In the conditions where the increment was varied and compared to a fixed decrement, the reciprocal was taken and averaged with the conditions where the decrement was varied. Figure 4 shows results averaged across participants. The gray circle indicates a ratio of 1 where perceived contrast is equivalent for increments and decrements.

The data show an asymmetry similar to that found in Experiment 1 (Figure 2), where +S cone colors appear to have higher contrast when paired with luminance increments than when paired with luminance decrements. However for these data, the opposite is true for the −S cone colors. These colors appear to have higher contrast when paired with decrements than when paired with increments. An ANOVA was performed comparing the increment over decrement sensitivities of the eight colors across participants. Adjustment of degrees of freedom according to the Greenhouse-Geisser correction was again used due to a violation of sphericity arising from unequal variances. A main effect of the increment/decrement sensitivities across colors, *F*_(1,3.42)_ = 4.86, *p* = 0.008, was found. A *post-hoc t*-test comparing the data from the +S cone and −S cone directions revealed a significant asymmetry in perceived contrast when isoluminant contours are paired with contours of luminance increments vs. decrements: *t*_(6)_ = 5.27, *p* = 0.002. While colors with +Scone input seem to require more of a luminance decrement to match its luminance increment counterpart in contrast, −S colors seem to be biased in the opposing direction, +L−M cone and −L+M colors seem to require roughly equal amounts of increment to decrement ratios to be contrast matched. The increase in perceived contrast for +S colors and luminance increments would predict an increase in the magnitude of the watercolor illusion for these colors as observed in Experiment 1.

## Discussion

While the current study focused on the role of S cone and achromatic contrast and contribution to the assimilative spreading of the watercolor illusion, other studies have shown strong hue shifts induced by high spatial frequency S cone patterns. Monnier and Shevell studied chromatic induction induced by thin rings, similar in spatial frequency to lines used for the watercolor illusion (Monnier and Shevell, 2004). Comparing the hue shift of rings on a uniform background to rings bordered by contrasting S cone patterns of rings, they found large assimilative hue shifts when the rings were bordered by other rings, compared to a smaller contrast effect when the rings were on uniform backgrounds. While the colored rings directly bordering the test ring caused an assimilative effect on the ring, the outer rings (similar to the outer contour in the watercolor illusion) caused a contrast effect. We here present a similar finding comparing results from our Experiments 2 and 3. In Experiment 2 we find that there is not a large shift in saturation when comparing a single chromatic contour to a single achromatic black or white contour on a uniform background. However in Experiment 3 we find a shift in the perceived contrast of chromatic and achromatic contours when they are patterned together.

Previous studies on the chromatic watercolor effect have reported that yellow produces weaker color spreading than other colors (Pinna et al., 2001; Devinck et al., 2005; Coia et al., 2014). Since the watercolor illusion is dependent on contrast of the two inducing contours, this implies that the yellows used in previous studies may have contrasted less than other colors. Coia et al. (2014) found an asymmetry in ±S (blue/yellow) illusion strengths even after equating for the perceived contrast of colors. However this equation of the perceived contrast was done in the presence of dark achromatic contours, and the results of Experiment 3 here reported that the apparent contrast produced when pairing −S (yellowish) colors with luminance increments and those produced when pairing with decrements are not equivalent. The difference in apparent contrast may partially explain the results of Experiment 1 of the present study.

Another way to equate the contrast of different unipolar colors would be to measure the amount of color required to match an opponent color of fixed contrast. By performing suprathreshold contrast matching comparing unipolar isoluminant colors to their opponent counterparts, Switkes (2008) found an asymmetry along an intermediate (blue/yellow) axis of color space showing that blue was less salient than yellow at equal cone contrasts. The direction of the asymmetry reported by Switkes (2008) is similar to the direction in which we find our greatest asymmetry in Experiments 1 and 3.

Vingrys and Mahon (1998) conducted experiments measuring unipolar threshold and discrimination mechanisms. They found asymmetries for blue/yellow (±S) and luminance increments and decrements, and concluded that the visual system has a lower threshold activity and higher suprathreshold sensitivity for decrements (yellow and black) than for increments (blue and white). No consistent asymmetry was found for red/green, similar to the current results. Additionally, they observed large masking effects when blue and white where presented in combination, while yellow showed minimal masking. A question they raise is whether the percept of yellow is mediated by blue-off cells or a reduction in the blue-on response. Differences in blue-on and blue-off pathways may also underlie the asymmetries reported here.

It has been a longstanding puzzle as to the underlying mechanisms that mediate the S opponent visual pathways that ultimately lead to our blue/yellow perception. The S cones themselves have been shown to receive feedback from horizontal cells, and two distinct types of bipolar cells receive on/off S input (Dacey et al., 2014). While previous single-cell recording studies have found S-off cells rather elusive, cells in the lateral geniculate nucleus of the thalamus (LGN) have been recorded which seem to combine S and M signals and contrast with L to produce an S off response (Tailby et al., 2008). It is possible that differences in receptive field size and/or different L to M cone weights of post receptoral S-on and S-off cells may be responsible for some of the asymmetries observed in psychophysical experiments (see Smithson, 2014). Future research may bridge the gap between these physiological/anatomical asymmetries in the S opponent pathway and psychophysical asymmetries found in humans.

The results of the current study largely agree with the results of previous studies showing the dependence of the magnitude of assimilative color spreading on contour contrast as well as studies (Devinck et al., 2006; Cao et al., 2011) that have shown the watercolor effect to depend on both contrast and polarity of contrast of the contours. The present results suggest that the mechanisms that produce the watercolor effect may not receive equally weighted inputs from the different color and luminance pathways. In addition, the asymmetries within the S-cone pathway responses suggest that on and off pathways may contribute unequally to the effect. Furthermore, there is a difference between perceptual and physical contrast of contours used to create watercolor spreading, where isoluminant contours with S cone input have asymmetric contrast effects when paired with luminance increment and decrement contours.

## Conflict of interest statement

The authors declare that the research was conducted in the absence of any commercial or financial relationships that could be construed as a potential conflict of interest.

## References

[B1] Brainard D. H. (1997). The psychophysics toolbox. Spat. Vis. 10, 433–436 10.1163/156856897x00357 9176952

[B2] Cao B. Yazdanbakhsh A. Mingolla E. (2011). The effect of contrast intensity and polarity in the achromatic watercolor effect. J. Vis. 11, 18 10.1167/11.3.18 21436347

[B3] Coia A. J. Jones C. Duncan C. S. Crognale M. A. (2014). Physiological correlates of watercolor effect. J. Opt. Soc. Am. A Opt. Image Sci. Vis. 31, A15–A22 10.1364/JOSAA.31.000A15 24695164

[B4] Dacey D. M. Crook J. D. Packer O. S. (2014). Distinct synaptic mechanisms create parallel S-ON and S-OFF color opponent pathways in the primate retina. Vis. Neurosci. 31, 139–151 10.1017/s0952523813000230 23895762 PMC4309572

[B5] Derrington A. M. Krauskopf J. Lennie P. (1984). Chromatic mechanisms in lateral geniculate nucleus of macaque. J. Physiol. 357, 241–265 6512691 10.1113/jphysiol.1984.sp015499 PMC1193257

[B6] Devinck F. Delahunt P. B. Hardy J. L. Spillmann L. Werner J. S. (2005). The watercolor effect: quantitative evidence for luminance-dependent mechanisms of long-range color assimilation. Vision Res. 45, 1413–1424 10.1016/j.visres.2004.11.024 15743611 PMC2614238

[B7] Devinck F. Hardy J. L. Delahunt P. B. Spillmann L. Werner J. S. (2006). Illusory spreading of watercolor. J. Vis. 6, 7 10.1167/6.5.7 16881793 PMC2583221

[B8] Devinck F. Knoblauch K. (2012). A common signal detection model accounts for both perception and discrimination of the watercolor effect. J. Vis. 12, 19 10.1167/12.3.19 22438468

[B9] Greenhouse S. W. Geisser S. (1959). On methods in the analysis of profile data. Psychometrika 24, 95–112 10.1007/bf02289823

[B10] MacLeod D. I. Boynton R. M. (1979). Chromaticity diagram showing cone excitation by stimuli of equal luminance. J. Opt. Soc. Am. 69, 1183–1186 10.1364/josa.69.001183 490231

[B21] Monnier P. Shevell S. K. (2004). Chromatic induction from S-cone patterns. Vision Res. 44, 849–856 10.1016/j.visres.2003.11.004 14992830

[B11] Pelli D. G. (1997). The VideoToolbox software for visual psychophysics: transforming numbers into movies. Spat. Vis. 10, 437–442 10.1163/156856897x00366 9176953

[B12] Pinna B. (1987). “Un effetto di colorazione,” in Il Laboratorio e la Città. XXI Congresso Degli Psicologi Italiani, eds Majer V. Maeran M. Santinello M. (Milano: Società Italiana di Psicologia), 158

[B13] Pinna B. (2004). The role of the Gestalt principle of similarity in the watercolor illusion. Spat. Vis. 18, 185–207 10.1163/1568568053320639 15856936

[B14] Pinna B. Brelstaff G. Spillmann L. (2001). Surface color from boundaries: a new ‘watercolor’ illusion. Vision Res. 41, 2669–2676 10.1016/s0042-6989(01)00105-5 11520512

[B15] Smithson H. E. (2014). S-cone psychophysics. Vis. Neurosci. 31, 211–225 10.1017/S0952523814000030 24759446

[B16] Switkes E. (2008). Contrast salience across three-dimensional chromoluminance space. Vision Res. 48, 1812–1819 10.1016/j.visres.2008.05.014 18602656

[B17] Switkes E. Crognale M. A. (1999). Comparison of color and luminance contrast: apples versus oranges? Vision Res. 39, 1823–1831 10.1016/s0042-6989(98)00219-3 10343874

[B18] Tailby C. Solomon S. G. Lennie P. (2008). Functional asymmetries in visual pathways carrying S-cone signals in macaque. J. Neurosci. 28, 4078–4087 10.1523/jneurosci.5338-07.2008 18400907 PMC2602833

[B19] Vingrys A. J. Mahon L. E. (1998). Color and luminance detection and discrimination asymmetries and interactions. Vision Res. 38, 1085–1095 10.1016/s0042-6989(97)00250-2 9666968

[B20] Weibull W. (1951). A statistical distribution function of wide applicability. J. Appl. Mech. 18, 293–297

